# The association between night shift work and elevated risk of smoking use among ride-hailing drivers: findings from a cross-sectional study

**DOI:** 10.3389/fpubh.2026.1739252

**Published:** 2026-07-09

**Authors:** Tenglong Yan, Jianjian Su, Binshuo Hu, Xin Song, Zhihui Wang, Siyuan Wang, Meili Gao, Hong Yu, Dongsheng Niu, Xiaoshun Wang, Rui Guan, Xiaowen Ding

**Affiliations:** 1 Beijing Prevention and Treatment Hospital of Occupational Disease, Beijing Institute of Occupational Disease Prevention and Treatment, Beijing, China; 2 The Third People's Hospital of Tianshui, Tianshui, China; 3 School of Public Health, Hangzhou Medical College, Hangzhou, China; 4 Beijing Rehabilitation Hospital, Capital Medical University, Beijing, China

**Keywords:** alcohol, long working hours, night shift, ride-hailing drivers, tobacco

## Abstract

**Background and objectives:**

Night shift work is linked to smoking in traditional occupations, while the association with substance use among ride-hailing drivers is unknown. This study examined the associations of night shift work with tobacco and alcohol use behaviors among ride-hailing drivers in China, a vulnerable and understudied occupational group.

**Methods:**

A cross-sectional study for ride-hailing drivers in Beijing, China (PWS study), was conducted and assessed night shift, smoking and drinking habits via structured questionnaires. Heavy smokers and high intensity drinking workers were further identified based on established standards. Multivariable logistic regression models were performed and adjusted for sociodemographic and occupational covariates (sex, age, income, etc.) to evaluate the associations of night shift work with tobacco and alcohol consumption habits.

**Results:**

Among 858 ride-hailing drivers, 23% worked night shift. Night shift workers exhibited significantly higher smoking prevalence (59.4% vs. 47.7%) and higher smoking index (0 vs. 140), compared with day shift workers. In the logistic regression model, night shift workers had significantly higher odds of being a smoker [(*OR* = 1.595 (95% *CI*, 1.108–2.307)], and a heavy smoker (smoking index ≥ 400; *OR* = 1.682, 95% *CI*: 1.002–2.824) compared with day shift workers. The study found no link between alcohol consumption and night shifts among ride-hailing drivers.

**Conclusion:**

The high smoking prevalence among ride-hailing drivers, further elevated by night shift work, highlights the urgent need for targeted interventions in China’s platform economy.

## 1 Introduction

The rise of night shift work, particularly night shifts, in the modern 24/7 economy has raised critical occupational health concerns. Night shift working affected approximately 20% of the global workforce, with night shifts disrupting circadian rhythms and exacerbating metabolic and cardiovascular risks (1), which is attributed 745,000 annual deaths to ischemic heart disease and stroke (2). In addition, a growing body of evidence has well-established that night shift work schedules can increase the risk of tobacco use and alcohol abuse (2, 3). Meta-analyses demonstrated that night shift workers exhibit 30% higher odds of smoking (4) and 25% greater alcohol consumption (5) compared to day schedule workers. Circadian misalignment from night shifts dysregulates dopamine and serotonin pathways, increasing susceptibility to addictive behaviors (6).

In China, the number of ride-hailing drivers has grown rapidly in recent years, reaching approximately 84 million individuals, with food delivery riders, couriers, and ride-hailing drivers being the most representative groups. This group faces unique occupational stressors due to algorithm-driven dispatch systems that reduce work breaks, increase task loads, and employment instability. Most individuals in this group are full-time workers in China. Driven by income instability, many of these workers turn to night shift work to secure higher earnings (7, 8), resulting to 1/5 of whom mainly worked at night. The previous study posited that night schedule workers had a preference for using tobacco and alcohol to combat fatigue and social isolation (9), yet this remained untested among ride-hailing drivers. Most evidence about night shift work derived from healthcare, manufacturing, or transportation sectors, leaving ride-hailing drivers, a group with distinct job precarity largely unexplored. Ride-hailing drivers face additional stressors, such as real-time performance monitoring and income volatility, which may amplify these risks of tobacco and alcohol abuse, and result in adverse health event (8). The constant mobility of ride-hailing drivers through urban environments grants them easy, ubiquitous access to tobacco and alcohol. This physical availability, coupled with the chronic stress induced by job insecurity and non-standard employment, significantly contributes to the development and reinforcement of tobacco and alcohol use habits (10–12).

For drivers in the platform economy, chronic use of these substances can impair cognitive function, judgment, and reaction time, elevating the risk of traffic accidents and occupational injuries (6). Beyond immediate safety hazards, sustained tobacco and alcohol consumption was strongly associated with the development of cardiovascular diseases, hypertension, metabolic disorders, and certain cancers, which may be exacerbated by the physiological stress induced by irregular night shift work and prolonged sitting (2, 3). Given that many ride-hailing drivers already endure extended working hours, economic precarity, and sleep disruption, the adoption of substance use as a coping strategy could accelerate a detrimental health cycle (13). This not only threatens individual well-being but also undermines public safety and imposes broader socioeconomic costs (14, 15). Therefore, targeted health education and supportive workplace interventions are essential to promote healthier stress-management alternatives and mitigate these preventable risks within this rapidly expanding workforce (16). However, no study to date has examined these associations among ride-hailing drivers, a group facing unique stressors such as algorithmic control and income volatility that may amplify substance use risks. We hypothesized that night shift ride-hailing drivers have a higher likelihood of tobacco and alcohol use. Hence, this study aimed to investigate the associations between night shift work and substance use behaviors (smoking and drinking) among ride-hailing drivers in Beijing, China, to address critical gaps in the literature for the ride-hailing drivers health effect.

## 2 Methods

### 2.1 Study design and participants

A cross-sectional study was conducted to evaluate the occupational risk factors and health status for ride-hailing drivers in Beijing, China, from 2024 to 2025, which was named as Chinese Platform Workers Study (PWS) (13, 17). The inclusion criteria were: (1) At least one year of continuous employment as full time platform workers (delivery riders, couriers, or ride-hailing drivers); (2) No significant medical records, including chronic respiratory, hepatic, or renal disease, other severe systemic illnesses, or major trauma requiring hospitalization; (3) Age between 18 and 60 years old (inclusive); (4) Voluntary participation with signed informed consent. The exclusion criteria were: (1) Pregnancy, lactation, or menopause (for female participants); (2) Difficulty communicating or inability to cooperate in completing the questionnaire. All the study participants were administered through a structured questionnaire and physical examinations. The sampling was random within this accessible population, but the underlying occupational gender segregation naturally resulted in a male-dominated sample. Assuming a conservative smoking prevalence (*p*) of 50% among ride-hailing drivers, a 95% confidence level (*Z* = 1.96), and a desired precision (*d*) of ±3.5%, the minimum required sample size was calculated as approximately 784 participants. Our achieved sample size of 858 exceeds this threshold, indicating adequate precision for prevalence estimation. The questionnaire was used to collect information on general demographics, lifestyle habits, occupational history, personal and family medical history, medication use, musculoskeletal disorders, and occupational stress (Supplementary Table S1). For this study, only ride-hailing drivers were included as the research participants. This study was conducted in accordance with the Declaration of Helsinki and was approved by the Institutional Review Board of the Beijing Institute of Occupational Disease Prevention and Control (Ethics Approval No. C2023003). All participants provided written informed consent prior to study participation.

### 2.2 Measurement of night shift work

Night shift work has been widely recognized as critical occupational risk factors associated with adverse health outcomes. In this study, we adopted standardized definitions to ensure consistency with prior epidemiological research. Night shift work was defined as employment schedules requiring work primarily between 22:00 and 05:00, in alignment with the circadian disruption window commonly used in occupational health studies (3, 18). In this study, participants working mainly worked between 10:00 p.m. and 5:00 a.m. were classified as the night shift group, and conversely, as the day shift group. This definition captures the period of biological night, during which melatonin secretion peaks and sleep propensity is highest, thus maximizing the misalignment between work demands and endogenous circadian rhythms (19). Given the pervasive nature of extended working hours within this occupational group, this study focused specifically on the impact of night shift work as the primary exposure factor (17).

### 2.3 Measurement of smoking and drinking habits

The smoking and high-intensity smoking were examined. The participants were questioned the current smoking habits, age of smoking initiation, daily cigarette consumption, and smoking cessation timeline, which was shown in Table 1. Participants were classified as smokers if they smoked at least one cigarette per day on average, and as non-smokers otherwise (20), the method had been verified to be feasible many times previously. Smokers were further categorized into heavy and light smokers, with heavy smoker defined as: smoking index > 400, while the smoking index was defined by the formula: (number of years smoking) × (average daily cigarette consumption) (21). Heavy smokers are generally recognized as individuals at a significantly higher risk of developing lung cancer (22). The drinking and high-intensity drinking were also examined. The participants were asked to report: frequency of alcohol consumption over the past 12 months and number of binge drinking episodes (if applicable) in the past 30 days. The methods of evaluating for drinking were mainly from the National Health and Nutrition Examination Survey (23, 24). Participants who consumed alcohol at least once per month on average were classified as current drinkers (24), while high-intensity drinking was defined as the regular consumption of more than 75 g of pure alcohol on a single occasion (Supplementary Table S1) (25).

**Table 1:** The demographics characteristics across work shift or working hours.

Characteristics	Day shift	Night shift	*P*
*n* (%)	661 (77.0)	197 (23.0)	-
Sex			0.149
Male	595 (90.0%)	184 (93.4%)	
Female	66 (10.0%)	13 (6.6%)	
Age	43.00 (37.00, 50.00)	41.00 (36.00, 47.00)	0.002 ^*^
Nationality			0.100
Han	621 (93.9%)	191 (97.0%)	
Other	40 (6.1%)	6 (3.0%)	
Household registration			0.148
City and town	472 (71.4%)	151 (76.6%)	
Country	189 (28.6%)	46 (23.4%)	
Educational status			0.467
Junior high school or below	285 (43.1%)	85 (43.1%)	
Senior high school	131 (19.8%)	32 (16.2%)	
College degree or above	245 (37.1%)	80 (40.6%)	
Marital status			0.500
Unmarried	50 (7.6%)	20 (10.2%)	
Married or cohabiting	565 (85.5%)	163 (82.7%)	
Divorced or widowed	46 (7.0%)	14 (7.1%)	
Number of family population	4.00 (3.00, 4.00)	4.00 (3.00, 4.00)	0.369
Personal monthly income			0.267
≤ 6,000	187 (28.3%)	42 (21.9%)	
6,001 ~ 8,000	175 (26.5%)	48 (24.4%)	
8,001 ~ 10,000	124 (18.8%)	44 (22.3%)	
> 10,000	175 (26.5%)	62 (31.5%)	
Current working age (years)	6.00 (3.00, 9.00)	6.00 (3.00, 8.00)	0.343

### 2.4 Other variables

In addition to the primary exposure and outcome variables, this study accounted for several sociodemographic and occupational covariates that may influence the observed associations. These variables were selected based on their established relevance in public health and occupational epidemiology literature. Participants were categorized into Han ethnicity or other ethnic groups based on ethnicity. The body mass index (BMI) was measured. Household registration type (Hukou) was classified as urban or rural, as prior studies have documented significant disparities in smoking and alcohol consumption rates between urban and rural populations in China (26). The Chinese household registration system divides citizens into two categories: urban and rural Hukou. This classification indicates the individual’s registered permanent residence area, which is officially documented by the government. Rural registration typically applies to individuals engaged primarily in agricultural production, who may also seek temporary employment in cities during non-farming seasons. In contrast, urban registration is designated for residents whose livelihoods are based on non-agricultural sectors, with employment and social services centered in towns and cities. Participants’ education levels were stratified as: (1) ≤ 9 years (compulsory education), (2) 10–12 years (high school/vocational training), and (3) ≥ 13 years (tertiary education), aligning with UNESCO’s International Standard Classification of Education. Personal monthly income was adjusted for given its association with health-seeking behaviors and occupational stress (41). Monthly personal income was categorized into: (1) ≤ 6,000 CNY, (2) 6,001–8,000 CNY, (3) 8,001–10,000 CNY, and (4) > 10,000 CNY based on China’s national wage distribution patterns. Age, household size, and work experience were obtained via direct participant self-report and treated as a continuous variable (measured in whole years) for all analytical purposes (27).

### 2.5 Statistical analysis

All the analyses were performed using R Statistical software (version 4.2.2; R Core Team, 2022) on Windows 10. Categorical variables (e.g., sex, household registration, educational status, smoking and drinking habits) were presented as counts and percentages (*n*, %). Continuous variables (e.g., age, smoking index) were first assessed for normality using the Shapiro–Wilk test. Normally distributed continuous variables were reported as mean ± standard deviation (*SD*), while non-normally distributed variables were reported as median with interquartile range (*IQR*). To compare demographic and behavioral characteristics between night shift and day shift workers, we used Pearson’s chi-square test (or Fisher’s exact test when expected cell counts < 5) for categorical variables. For continuous variables, the independent two-sample *t*-test was applied for normally distributed data, and the Mann–Whitney U test was used for non-normally distributed data. To examine the independent associations between night shift work (exposure variable, dichotomous: night shift vs. day shift) and substance use outcomes (smoking status, heavy smoking status, drinking status, and high-intensity drinking), we performed multivariable logistic regression analyses. Covariates were selected based on established literature and included: sex, age (continuous), body mass index (BMI, continuous), educational level (categorical), and personal monthly income (categorical). All covariates were entered into the model simultaneously (enter method). Results were reported as odds ratios (*OR*) with 95% confidence intervals (*CI*). Model fit was assessed using the Hosmer-Lemeshow goodness-of-fit test. Prior to fitting the multivariable logistic regression models, multicollinearity among the independent variables was assessed using the variance inflation factor (VIF). A VIF value exceeding 5 or 10 was considered indicative of problematic collinearity (or a tolerance value < 0.2 or < 0.1). No variable exclusion or data transformation was performed if all VIF values were below the predetermined threshold. All hypothesis testing employed two-sided comparisons, with statistical significance defined *a priori* at *p* < 0.05.

## 3 Results

### 3.1 The demographics characteristics for participants

The demographic characteristics of the ride-hailing drivers stratified by work shift schedule (day shift vs. night shift) are presented in Table 2. 858 participants were enrolled in this study, with 661 (77.0%) working the day shift and 197 (23.0%) working the night shift. There was no statistically significant difference in sex distribution between the shift pattern (*p* = 0.149), with males comprising 90.0% of the day shift and 93.4% of the night shift. Night shift workers were significantly younger than day shift workers (median age: 41.00 vs. 43.00 years, *p* = 0.002). The majority of participants were of Han ethnicity (93.9% in day shift and 97.0% in night shift), with no significant differences across shifts. Household registration revealed a higher proportion of urban residents in the night shift (76.6%) compared to the day shift (71.4%), though this difference was not statistically significant (*p* = 0.148). No significant differences were observed in current working age across work shift pattern.

**Table 2:** The demographic characteristics across work shift patterns [*n* (%) or *M* (*IQR*)].

Characteristics	Day shift	Night shift	*P* ^a^
*n* (%)	661 (77.0)	197 (23.0)	-
Sex			0.149
Male	595 (90.0%)	184 (93.4%)	
Female	66 (10.0%)	13 (6.6%)	
**Age** (years old)	43.00 (37.00, 50.00)	41.00 (36.00, 47.00)	0.002 ^* b^
Nationality			0.100
Han	621 (93.9%)	191 (97.0%)	
Other	40 (6.1%)	6 (3.0%)	
Household registration			0.148
City and town	472 (71.4%)	151 (76.6%)	
Country	189 (28.6%)	46 (23.4%)	
BMI			0.278
≤ 23.9 kg/m^2^	102 (15.4%)	30 (15.2%)	
24.0 ~ 27.9 kg/m^2^	277 (41.9%)	71 (36.0%)	
≥ 28 kg/m^2^	282 (42.7%)	96 (48.7%)	
Educational status			0.467
Junior high school or below	285 (43.1%)	85 (43.1%)	
Senior high school	131 (19.8%)	32 (16.2%)	
College degree or above	245 (37.1%)	80 (40.6%)	
Marital status			0.500
Unmarried	50 (7.6%)	20 (10.2%)	
Married or cohabiting	565 (85.5%)	163 (82.7%)	
Divorced or widowed	46 (7.0%)	14 (7.1%)	
Number of family population (*n*)	4 (3, 4)	4 (3, 4)	0.369
Personal monthly income			0.267
≤ 6,000	187 (28.3%)	42 (21.9%)	
6,001 ~ 8,000	175 (26.5%)	48 (24.4%)	
8,001 ~ 10,000	124 (18.8%)	44 (22.3%)	
> 10,000	175 (26.5%)	62 (31.5%)	
Current working age (years)	6 (3, 9)	6 (3, 8)	0.343
≤ 5 years	303 (46.0)	93 (47.2)	0.775
> 5 years	355 (54.0)	104 (52.8)	

*P* < 0.05; ^a^Chi-square test; ^b^rank sum test.

### 3.2 The tobacco and alcohol consumption habits

The consumption patterns of tobacco and alcohol among all participants, stratified by shift schedule are presented in Table 3. Night shift workers exhibited a higher prevalence of smoking (59.4%) compared with day shift workers (47.7%, *p* = 0.004). The median smoking index was notably higher among night shift workers (140.00) than day shift workers (0.00, *p* = 0.003). The percentage of heavy smoking for night shift workers (13.7%) was slightly higher than that for day shift workers (10.9%) although without significant difference. No significant differences were detected in drinking habits between day and night shift workers (*p* = 0.256). The proportion of participants who consumed alcohol was slightly higher among night shift workers (56.3%) compared with day shift workers (51.7%), while the difference was not statistically significant.

**Table 3:** The tobacco and alcohol consumption habits across work shift pattern [*n* (%) or *M* (*IQR*)].

Variables	All	Day shift	Night shift	*P* ^a^
Smoking habits				0.004^*^
No	426 (49.5)	346 (52.3%)	80 (40.6%)	
Yes	432 (50.5)	315 (47.7%)	117 (59.4%)	
Smoking index	0 (0, 270)	0 (0, 250)	140 (0, 300)	0.003 ^*,b^
Heavy smoker	99 (11.5)	72 (10.9%)	27 (13.7%)	0.278
Drinking habits				0.256
No	405 (47,2)	319 (48.3%)	86 (43.7%)	
Yes	453 (52.8)	342 (51.7%)	111 (56.3%)	
High intensity drinking				0.346
No	628 (73.2)	611 (92.4%)	178 (90.4%)	
Yes	69 (8.0)	50 (7.6%)	19 (9.6%)	

^*^P < 0.05; ^a^Chi-square test; ^b^rank sum test.

### 3.3 The associations of night shift work with smoking and drinking habits

**Figure 1:**
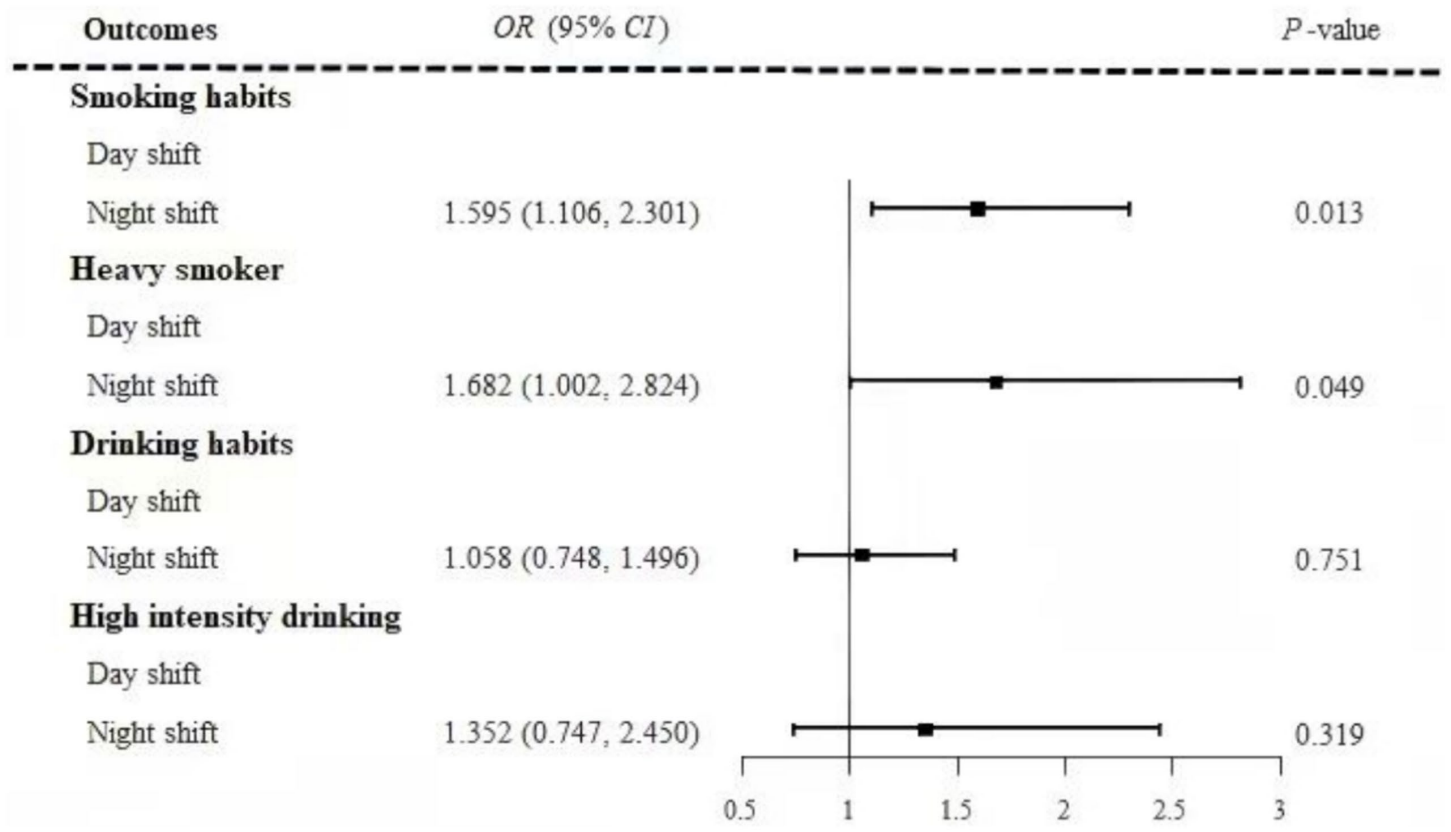
The associations of night shift with smoking and drinking habits.

The associations between night shift work and smoking/drinking habits are presented in Figure 1. The variables for sex, age, nationality, household registration, BMI, educational status, marital status, number of family population, monthly personal income, current working age, and weekly working hours were adjusted in the logistic models. Multicollinearity diagnostics revealed that all variance inflation factor (*VIF*) values for the independent variables included in the regression models were below 2, indicating no evidence of problematic collinearity among the covariates. Compared with the day shift workers, the night shift workers exhibited significantly higher odds of smoking habits (*OR* = 1.595, 95% *CI*: 1.108–2.307, *p* = 0.013) and heavy smoking (*OR* = 1.682, 95% *CI*: 1.002–2.824, *p* = 0.049), which demonstrated that the night shift workers were more significantly to consume tobacco. Furthermore, we tested the interaction between night shift work and salary, and current working experience by including their product term in the regression model for the smoking habits and heavy smoker, while the analysis did not reveal a statistically significant interaction. However, no significant associations were observed between night shift work and current drinker (*OR* = 1.058, 95% *CI*: 0.748–1.498, *p* = 0.751) or high intensity drinking (*OR* = 1.352, 95% *CI*: 0.733–2.420, *p* = 0.319).

## 4 Discussion

This study provides novel evidence on the synergistic effects of night shift work on smoking behaviors among Chinese ride-hailing drivers, a vulnerable population in the rapidly expanding internet economy. Two key findings emerged: (1) Night shift work was associated with a higher prevalence of smoking among ride-hailing drivers; (2) While drinking habits were comparable between night-shift and day-shift workers, these findings underscore a specific need for tobacco control interventions among night shift ride-hailing drivers, informing future health measures tailored to this group.

Our findings revealed that ride-hailing drivers in China face disproportionately high rates of night shifts (23.0%), exceeding prevalence estimates from traditional sectors like healthcare (15–20% night shift schedule) (2, 3). The smoking prevalence among night shift ride-hailing drivers (59.4%) was markedly higher than in Chinese general population (26.6%) (26) and other occupational groups (e.g., 36.8% in healthcare workers) (4). Similarly, alcohol use (56.3% in night shift workers) surpassed national averages (35.5%) (8), though differences were non-significant, possibly due to China’s strict enforcement of laws against drunk driving (6). This disparity underscores the unique vulnerability of ride-hailing drivers, who face compounded stressors from algorithmic control, income instability, and lack of labor protections (27).

Our finding that night shift workers had 1.595 - fold higher smoking odds aligns with meta-analytic data (*OR* = 1.3–1.5) from healthcare and manufacturing sectors (9). However, the magnitude of risk among ride-hailing drivers with both night shifts and LWH (*OR* up to 3.9) exceeded previous reports, possibly reflecting the compounding effects of job insecurity and algorithmic control unique to platform work (28, 29). The lack of association with alcohol use contrasts with European cohort studies (30), suggesting cultural differences in coping mechanisms, Chinese workers may prioritize smoking due to its social acceptability and immediate stress relief (31, 32). The compensatory hypothesis may explain the results: ride-hailing drivers face triple stressors, such as circadian misalignment, income volatility, and real-time performance monitoring, potentially driving tobacco use as a maladaptive coping strategy (27). Neurobiologically, chronic night shifts impair prefrontal cortex function (33), reducing impulse control over smoking, while long working hours that induced cortisol dysregulation may enhance nicotine reward sensitivity (34).

The elevated risks of smoking associated with night shifts among ride-hailing drivers necessitate comprehensive interventions that integrate labor policy reforms, technological innovations, and public health strategies. Current evidence suggests that regulatory measures should prioritize enforcing working-hour limits through platform embedded monitoring systems, as demonstrated by the EU Directive on Transparent and Predictable Working Conditions, which mandates rest periods and algorithmic transparency. From a technological perspective, modifying dispatch algorithms to reduce consecutive night shift, represents a feasible intervention to mitigate circadian disruption. Digital health solutions, particularly Just-In-Time Adaptive Interventions (JITAIs) that deliver behavioral nudges through worker apps (35). These multi-level strategies must be tailored to local labor contexts while drawing on global best practices to effectively address the unique health challenges faced by ride-hailing drivers in the internet economy. In addition, smoking is a complex behavioral habit influenced by a multitude of demographic and sociological factors beyond night-shift work. Sociodemographic characteristics of sex, household registration, educational status, marital status, and salary were all associated with tobacco consumption habits. In China, individuals with rural hukou often exhibit higher smoking rates than their urban counterparts. This is linked to several factors, including less exposure to comprehensive tobacco control policies, targeted marketing by tobacco companies, and norms within rural social networks (36). Higher education was associated with greater health literacy, stronger perceived self-efficacy to quit, and social environments less conducive to smoking (37). Lower income was consistently associated with higher smoking prevalence and greater difficulty in quitting. This relationship is driven by factors such as financial stress, targeted marketing in lower-income communities, and the use of smoking as a coping mechanism for daily stressors (38). Comprising largely males with rural household registration, lower education levels, and limited income, this platform worker demographic was subject to intersecting structural inequalities that significantly increase their vulnerability to tobacco use. When designing interventions to reduce tobacco and alcohol use among this population, it is essential to comprehensively account for these sociological determinants.

Unlike the significant association observed for smoking, our study found no significant link between night shift work and alcohol consumption among ride-hailing drivers. This null finding warrants careful interpretation from several perspectives. First, China’s stringent drunk driving laws may serve as a powerful deterrent. Unlike smoking, which can be done discreetly during breaks, alcohol consumption impairs driving ability and carries severe legal consequences, including license suspension, fines, and even criminal liability. Ride-hailing drivers, who face real-time monitoring and customer ratings, may be particularly sensitive to these risks. Second, the nature of platform work, continuous driving with unpredictable passenger requests—may not easily accommodate alcohol use, whereas smoking can be done during short waiting times or between rides. Third, cultural factors may play a role. In Chinese occupational settings, smoking is often used as a quick stress-relief tool and social bonding activity, while alcohol consumption typically occurs after work hours or during meals, making it less directly linked to on-the-job coping mechanisms. Fourth, the high prevalence of smoking (59.4% among night shift workers) may leave less additional variance to be explained by alcohol use, given that individuals may rely predominantly on one substance as a primary coping strategy. Finally, our assessment of alcohol consumption (any use vs. non-use) may have been too broad to capture nuanced patterns. Future studies should examine specific drinking contexts (e.g., after-work vs. during breaks) and dose–response relationships to better understand the conditions under which night shift work might influence alcohol use. Additionally, smoking, alcohol use, and gambling frequently coexist, especially among individuals with lower socioeconomic status (32, 39, 40), where addictive behaviors tend to be more prevalent. In the specific population we are concerned with, the rates of smoking and drinking exceed the general population average.

This study has several main limitations. First, as a cross sectional study, our analysis cannot establish causal relationships between night shift work and adverse behavioral outcomes. Second, while we assessed current exposure to night shift work, we lacked data on cumulative exposure duration (e.g., years of night shifts or prolonged work schedules), which may play a critical role in health effects. Future longitudinal studies with detailed occupational histories are needed to address these gaps. Third, the underrepresentation of female ride-hailing drivers is a key limitation of our study, which may affect the generalizability of the results, particularly regarding the patterns and potential drivers of substance use. The future studies should make targeted efforts to recruit a larger and more balanced sample of female ride-hailing drivers to allow for gender-stratified analyses and more comprehensive conclusions. Fourth, this study did not examine psychosocial factors such as job control or mental health, which may mediate substance use behaviors. Future research should integrate these dimensions to clarify the pathways between night shifts and health outcomes among ride-hailing drivers.

A major strength of our study was the detailed assessment of night shift work’s association with tobacco and alcohol use among a specifically vulnerable occupational group. Our findings should be interpreted within the distinct socio-occupational context of ride-hailing drivers in China. The elevated smoking risk associated with night shift work is likely amplified by the confluence of several population-specific factors. Firstly, the demographic profile, predominantly male, with a substantial proportion holding rural household registration and having lower educational attainment, aligns with subgroups known to have higher baseline smoking prevalence and less access to tobacco control resources in China. Secondly, the pervasive job precarity, algorithmic management, and income volatility inherent to platform work create a chronic stress environment. Night shift work, superimposed on these existing stressors, may act as a critical tipping point, making tobacco use a more accessible, albeit maladaptive, coping mechanism. This study contributes novel evidence that for ride-hailing drivers, interventions targeting smoking must extend beyond individual behavior change or generic shift schedule regulations. Effective strategies need to be multi-level, addressing the intersection of unconventional working hours (like night shifts), the unique pressures of the platform economy, and the underlying socioeconomic determinants of health that characterize this workforce.

## Data Availability

The original contributions presented in the study are included in the article/Supplementary material, further inquiries can be directed to the corresponding author/s.
