# Supplementary material for: The association between night shift work and elevated risk of smoking use among ride-hailing drivers: findings from a cross-sectional study
Source: Front Public Health. 2026 Jul 9;14:1739252. doi: 10.3389/fpubh.2026.1739252 (PMC13391909; doi:10.3389/fpubh.2026.1739252)
Supplement: Supplementary file 1 [file Table_1.DOCX]

| **Table S1. The questionnaire** | |
| --- | --- |
| Basic Information | |
| 1. Demographic information | |
| 1.1 Name |  |
| 1.2 Gender | □ Male □ Female |
| 1.3 Date of Birth | 1. Year Month Day |
| 1.4 Your ethnicity | □ Han ethnic group □ Other ethnic groups (Fill in the specific ethnic minority) |
| 1.5 Household registration type | □ Urban household registration □ Rural household registration |
| 1.6 Mobile phone number | □ □ □ □ □ □ □ □ □ □ □ |
| 1.7 Your level of education | □ Elementary school and below □ Graduated from junior high school  □ High school / vocational school / technical school education  □ Associate degree graduation □ Bachelor's degree graduation  □ Postgraduate and above |
| 1.8 Your current **marital** status | □ Unmarried □ Married □ Cohabitation □ Separate living  □ Divorce □Widowed |
| 1.9 Type of medical insurance (multiple choice) | □ Urban and Rural Residents Basic Medical Insurance (new rural cooperative medical system or urban residents' medical insurance) □ Employee Medical Insurance  □ Commercial health insurance □ Public medical care □ No medical insurance |
| 1.10 Number of permanent residents in the family | A total of people，there are a total of children. |
| 1.11 **Individual** monthly income | □ ≤ 4000￥ □4001~6000￥ □6001~8000￥ □8001~10000￥ □> 10000￥ |
| 1.12 What are your **primary** sources of income? (Select up to 2 options) | □ Labor income □ Property income □ Retirement pension  □ Support from other family members □ Minimum living allowance  □ Others (business insurance benefits, unemployment living expenses, early retirement living expenses, etc.) |
| 1.13 How long have you been here locally? | □ Less than 1 year □ 1 to 2 years □ 2 to 3 years □ 4 to 5 years  □ More than 5 years |
| 1.14 Sleep duration | Generally, go to sleep at PM and wake up at AM, totaling hours of sleep. |
| 1.15 Do you often suffer from insomnia? | □ 3 times a week or more □ 1-2 times a week □ 1 to 3 times a month □Never |
| 1. Career history | |
| 2.1 What is your profession? | □ Ride-hailing driver □ Delivery rider □ Courier  □ Others__________(Fill in the specific occupation) |
| 2.6 Is this job full-time or part-time? | □ Full-time □ Part-time |
| 2.2 When did you **start working**? | ______________,______________ |
| 2.3 When did you **start working in this position**? | ______________,______________ |

| 2.3 Workload | | | The total duration of online order receiving or delivery each day is hours.  The daily travel distance is kilometers.  The daily number of orders received/delivered is orders. |
| --- | --- | --- | --- |
| 2.4 Working hours | | | Work an average of _____ days a week; Work an average of ______hours a day;  There are ______ breaks during work every day, with an average of ______minutes for each break. |
| 2.5 Do you often deliver orders/passengers at night (from 11:00 PM to 5:00 AM)? | | | □ Yes □ No |
| 2.6 What are the reasons for you changing jobs or coming to Beijing? **(Multiple choices are allowed)** | | | □ The salary is somewhat better □ Closer to family □ Introduced by a friend □ No better option. □ Other ______________ (please specify the reason) |
| 2.7 What is this job for you since coming to Beijing? | | | □ First job □ 2^nd^-3^rd^ job □ 4^th^-5^th^ job □ 6^th^ job and beyond |
| 2.8 If the accidental injury of personnel or vehicles is defined as a traffic accident, how many traffic accidents have occurred since engaging in this work? | | | □ 0 times □ Once □ 2 to 3 times □ More than 3 times |
| 3. Smoking | | | |
| 3.1 Do you smoke **now**? | | □ Yes, I smoke every day. □ Yes, but not every day.  □ Used to smoke, but I don't smoke now.🡺 **Jump to 3.4**  □ Never smoke🡺 **Jump to 4.1** | |
| 3.2 When did you start smoking **every day**? **The investigator noted: "If you can't remember, fill in '-9'"** | | □ □ years old | |
| 3.3 How many manufactured cigarettes do you smoke on average each day (week)?  **Investigator Note: Daily smokers answer option 1 for question 3.1, non-daily smokers answer option 2.** | | □□ cigarettes per day  □□ cigarettes per week  □ Do not smoke manufactured cigarettes. | |
| 3.4 Your time of quitting smoking (completely not smoking) | | □ □ □ □, □ □ | |
| 4. Drinking alcohol | | | |
| 4.1 Have you consumed alcohol in the past 12 months? | | □ Drank alcohol within 30 days  □ Drunk alcohol in the past 30 days.🡺 **Jump to 4.3**  □ Never drank alcohol🡺 **Jump to 5.1** | |

| 4.2 In the past 30 days, have you consumed more than 150 grams of strong liquor in one sitting, or more than 200 grams of light liquor, or more than 3.5 bottles of beer, or more than 6 cans of beer? | | □ □ times | | | | |  |
| --- | --- | --- | --- | --- | --- | --- | --- |
| 4.3 How often do you generally drink alcohol in the past 12 months? | | □ Every day □ 5-6 times per week □ 3-4 times per week  □ 1-2 times per week □ 1-3 times per month □ Less than once a month | | | | |  |
| 5. Dietary situation | | | | | | |  |
| 5.2 Is your meal schedule regular, and how many times do you eat? | | □ Regular meals, meals a day.  □ Irregular meal times, sometimes skipping a meal. | | | | |  |
| 5.3 What time do you usually have breakfast? | | □ □:□ □ (24-hour format) | | | | |  |
| 5.4 What time do you usually have dinner? | | □ □:□ □ (24-hour format) | | | | |  |
| 5.5 How often do you have breakfast each week? | | □ Every day □ 4-6 days/week □ 1-3 days/week □ Hardly | | | | |  |
| 5.6 How often do you eat or have a snack **after 9 PM** each week? | | □ Every day □ 4-6 days/week □ 1-3 days/week □ Hardly | | | | |  |
| 5.7 Do you have a habit of eating takeout? | | □ Every day □ 4-6 days/week □ 1-3 days/week □ Hardly | | | | |  |
| 5.8 What kinds of food did you eat **yesterday**? | | □ Tuber crops (such as corn, potatoes, sweet potatoes, etc.) □ Vegetables □ Fruits  □ Poultry and livestock meat □ Aquatic products □ Eggs  □ Milk and dairy products □ Soybeans and nuts □ Oil (including cooking oil) | | | | |  |
| 6. Medical history and family history | | | | | | |  |
| **6.1 Do you have any of the following diseases or have you received any of the following treatments?** | | | | | | |  |
| **Disease name** | **Are you sick?** | | | **Diagnosis time** | | **Are you taking medication?** |  |
| Hypertension | □ Yes □ No | | | The year □□□□ □ Not clear | | □ Yes □ No |  |
| Diabetes | □ Yes □ No | | | The year □□□□ □ Not clear | | □ Yes □ No |  |
| Hyperlipidemia | □ Yes □ No | | | The year □□□□ □ Not clear | | □ Yes □ No |  |
| Arrhythmia | □ Yes □ No | | | The year □□□□ □ Not clear | | □ Yes □ No |  |
| Valvular disease | □ Yes □ No | | | The year □□□□ □ Not clear | | □ Yes □ No |  |
| Angina pectoris | □ Yes □ No | | | The year □□□□ □ Not clear | | □ Yes □ No |  |
| Chronic kidney disease | □ Yes □ No | | | The year □□□□ □ Not clear | | □ Yes □ No |  |
| Myocardial infarction | □ Yes □ No | | | The year □□□□ □ Not clear | | □ Yes □ No |  |
| Coronary Intervention | □ Yes □ No | | | The year □□□□ □ Not clear | | □ Yes □ No |  |
| Bridge surgery | □ Yes □ No | | | The year □□□□ □ Not clear | | □ Yes □ No |  |
| Stroke | □ Yes □ No | | | The year □□□□ □ Not clear | | □ Yes □ No |  |
| Heart failure | □ Yes □ No | | | The year □□□□ □ Not clear | | □ Yes □ No |  |
| Cancer (excluding skin cancer) | □ Yes □ No | | | The year □□□□ □ Not clear | | □ Yes □ No |  |
| 7. Family history | | | | | | | |
| **7.1 Among your father, son, and sibling, is there anyone who has had the following diseases before the age of 55? [Multiple choices allowed]** | | | □ Hypertension □ Coronary heart disease □ Ischemic stroke (cerebral infarction)  □ Hemorrhagic stroke (cerebral hemorrhage) □ Diabetes □ Malignant tumor  □ Hypercholesterolemia □ Myocardial infarction □ None of the above | | | | |
| **7.2 Has anyone among your mother, daughter, and siblings had any of the following diseases before the age of 55? [You may select multiple options]** | | | □ Hypertension □ Coronary heart disease □ Ischemic stroke (cerebral infarction)  □ Hemorrhagic stroke (cerebral hemorrhage) □ Diabetes □ Malignant tumor  □ Hypercholesterolemia □ Myocardial infarction □ None of the above | | | | |
| **8.** Exercise | | | | | | |  |
| 8.1 How many times do you participate in physical exercise on average **per week**? (Single choice) | | | | | □ Almost every day □ 3 to 5 times □ 1 to 2 times  □ Never | |  |
| 8.2 Exercise after work (each session exceeding 30 minutes or causing sweating) | | | | | □ Never □ 1 to 3 times per quarter  □ 2 to 3 times a month □ 1 to 2 times a week  □ 3 times a week or more | |  |
| 8.3 How long do you usually spend on physical exercise **each week** on average? | | | | | □□ Hours/Week | |  |
| 8.4 How long do you spend **on average each day** in your spare time looking at your phone/ watching TV/ reading books or newspapers/ playing cards, etc.? | | | | | □□ Hours/Days | |  |
| 8.6 Compared to the past, has your weight changed significantly **in the past year**? | | | | | □ Not much has changed compared to before.  □ At least gained 5 pounds. □ At least lost 5 pounds. | |  |
| 8.7 **In the past year**, have you taken measures such as dieting or medication to lose weight? | | | | | □ Yes □ No | |  |
| 8.8 What was your weight approximately around the age of 25? | | | | | □ □ □ pounds □ Not clear | |  |

| **9. Job content scale, please answer according to your actual situation.** | | | | | |
| --- | --- | --- | --- | --- | --- |
| 9.1 | Work requires effort. | Hardly ever | Occasionally will | Often will | Always will |
| 9.2 | Overtime is required at work. | Hardly ever | Occasionally will | Often will | Always will |
| 9.3 | There is enough time to complete the work. | Hardly ever | Occasionally will | Often will | Always will |
| 9.4 | Doing conflicting things according to the different demands of others. | Hardly ever | Occasionally will | Often will | Always will |
| 9.5 | The job requires a certain speed of work. | Hardly ever | Occasionally will | Often will | Always will |
| 9.6 | How to carry out work, I have the decision-making power. | Hardly ever | Occasionally will | Often will | Always will |
| 9.7 | In work, many things can be decided by oneself. | Hardly ever | Occasionally will | Often will | Always will |
| 9.8 | Regarding what happens at work, one's own opinions have influence. | Hardly ever | Occasionally will | Often will | Always will |
| 9.8 | The job requires learning new knowledge or skills. | Hardly ever | Occasionally will | Often will | Always will |
| 9.10 | Work needs to be innovative. | Hardly ever | Occasionally will | Often will | Always will |
| 9.11 | The work is highly repetitive. | Not high | A bit high | High | Very high |
| 9.12 | Job Technical Requirements | Not high | A bit high | High | Very high |
| 9.13 | At work, you can do various different things. | Hardly can | Occasionally can | often can | Always can |
| 9.14 | Opportunities to utilize one's strengths in work. | Hardly can | Occasionally can | often can | Always can |
| 9.15 | Leaders can care about the welfare of their subordinates. | Hardly can | Occasionally can | often can | Always can |
| 9.16 | The leader is able to listen to their own opinions. | Hardly can | Occasionally can | often can | Always can |
| 9.17 | Leaders can help their subordinates carry out their work. | Hardly can | Occasionally can | often can | Always can |
| 9.18 | The leader is very organized, enabling subordinates to work together cohesively. | Hardly can | Occasionally can | often can | Always can |
| 9.19 | Colleagues are capable of doing their jobs. | Hardly can | Occasionally can | often can | Always can |
| 9.20 | Colleagues can help oneself at work. | Hardly can | Occasionally can | often can | Always can |
| 9.21 | Colleagues can care about themselves. | Hardly can | Occasionally can | often can | Always can |
| 9.22 | Colleagues' attitude towards oneself | Very unfriendly | Unfriendly | friendly | Very friendly |

| 10. Musculoskeletal Disorders Section | | | | | |
| --- | --- | --- | --- | --- | --- |
| 10.1 Have you been diagnosed by a doctor with any of the following conditions **(multiple choices are allowed)**? | | □ Chronic tendonitis of the wrist □ Eagle's beak diverticulitis  □ Carpal tunnel syndrome □ Epicondylitis □ Meniscus injury  □ Prepatellar bursitis □ None | | | |
| 10.2 Have you ever injured any of the following areas in an accident? **(You can select multiple options)** | | □ None □ Neck □ Shoulder □ Upper back □ Lower back (waist) □ elbow  □ Wrist/Hand □ Hip/Thigh □ Knee □ Ankle/Foot | | | |
| **10.3 Survey on 'Pain or Discomfort' in the Musculoskeletal System** | | | | | |
| 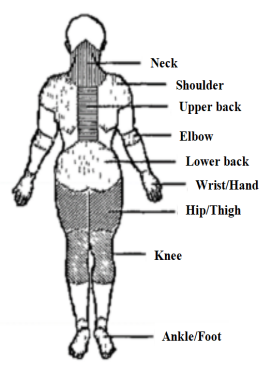 | Have you experienced any pain or discomfort **in the past year**? | | Write down the corresponding score based on the level of pain or discomfort. | The duration of symptoms such as pain each time they occur. | The frequency of symptoms such as pain |
|  | Tick the appropriate box; **If you choose 'No', do not answer the three questions on the right.** | | **0~10 points**  (0 is no pain, 10 is the worst pain) | ① Reach or exceed 1 month  ② Reach or exceed 1 week  ③ Reach or exceed 1 day  ④ Less than 1 day  ⑤ Never happened | ① Almost every day  ② Once a week  ③ 1 to 2 times each month  ④ 1 to 2 times each quarter  ⑤ 1 to 2 times a year |
| Neck | ① Yes ② No | |  | ① ② ③ ④ ⑤ | ① ② ③ ④ ⑤ |
| Shoulder | ① Yes ② No | |  | ① ② ③ ④ ⑤ | ① ② ③ ④ ⑤ |
| Upper back | ① Yes ② No | |  | ① ② ③ ④ ⑤ | ① ② ③ ④ ⑤ |
| Lower back (waist) | ① Yes ② No | |  | ① ② ③ ④ ⑤ | ① ② ③ ④ ⑤ |
| elbow | ① Yes ② No | |  | ① ② ③ ④ ⑤ | ① ② ③ ④ ⑤ |
| Wrist/Hand | ① Yes ② No | |  | ① ② ③ ④ ⑤ | ① ② ③ ④ ⑤ |
| Hip/Thigh | ① Yes ② No | |  | ① ② ③ ④ ⑤ | ① ② ③ ④ ⑤ |
| Knee | ① Yes ② No | |  | ① ② ③ ④ ⑤ | ① ② ③ ④ ⑤ |
| Ankle/Foot | ① Yes ② No | |  | ① ② ③ ④ ⑤ | ① ② ③ ④ ⑤ |
